# A Systematic Review of the Long-Term Clinical Success of Gastric Peroral Endoscopic Myotomy for Refractory Gastroparesis

**DOI:** 10.7759/cureus.39709

**Published:** 2023-05-30

**Authors:** Tamara Stojilkovic, Kelsey Staudinger, Jennifer Dennis

**Affiliations:** 1 Department of Medicine, Kansas City University, Kansas City, USA; 2 Department of General Surgery, Swedish Medical Center, Englewood, USA; 3 Department of Pathology & Anatomical Sciences, Kansas City University, Kansas City, USA

**Keywords:** gastric peroral endoscopic myotomy, gcsi, gpoem, gastric emptying, gastroparesis

## Abstract

Gastroparesis is a chronic and debilitating gastrointestinal disorder with few medical treatment options. Traditional surgical management has involved laparoscopic pyloromyotomy or gastric stimulation. In recent years, gastric peroral endoscopic myotomy (GPOEM) has become an attractive, less invasive option for patients with refractory gastroparesis. There is little information on the long-term clinical success of GPOEM in patients with refractory gastroparesis. This systematic review aims to evaluate the data on this procedure's long-term clinical efficacy and safety. A comprehensive literature review was done in PubMed, EMBASE, Ovid, and Google Scholar databases from the date of earliest entry in May 2017 up to August 15, 2022. The Gastroparesis Cardinal Symptom Index (GCSI) score, adverse reaction, and length of stay were analyzed. Eleven studies were eligible for inclusion (900 patients), seven of the studies were retrospective, while four were prospective. The GCSI is a 6-point Likert scale questionnaire that assesses improvement in gastroparesis. An average decrease of GCSI by 1 point compared to baseline GCSI for all patients (described as clinical success) was found in 662 patients out of 713 (92.8%) at one-year follow-up, 421 out of 460 (91.5%) at two-year follow-up, 270 out of 270 (100%) at three-year follow-up, and 102 out of 102 (100%) at four-year follow-up. Adverse events occurred in 62 out of 835 patients (in nine studies), with two of the most frequent being bleeding and mucosal tears. GPOEM is an effective and safe treatment option for patients with refractory gastroparesis, with symptom improvement noted up to four years postoperatively.

## Introduction and background

Gastroparesis is characterized by delayed gastric emptying in the absence of mechanical obstruction. This is a chronic disease in which the most common symptoms include nausea, early satiety, postprandial fullness, upper abdominal pain, and vomiting. The three main etiologies consist of idiopathic, diabetic, and post-surgical, which comprise most of all cases. In some cases, the symptoms can be debilitating, leading to a decrease in the quality of life and a financial burden with frequent hospital visits [1]. Gastroparesis affects more than 10 million patients, approximately three percent of the United States population [2]. The majority of these patients are women who are affected [1].

Treatment is started conservatively with dietary medications. If refractory and the symptoms persist, medical treatment includes medications that are prokinetic and antiemetic. Other options are Botox injections (no longer recommended by the American College of Gastroenterologists) or the placement of a gastric electrical stimulator [3]. If oral intake is intolerable and the patient is malnourished, enteral nutrition is necessary, most often through a jejunostomy tube. If those do not work, the next option is a laparoscopic pyloroplasty. All previous treatments have had limited success [4].

Gastric peroral endoscopic myotomy (G-POEM) is a more recent option that is a minimally invasive surgery with promising results. During the G-POEM procedure, an endoscope (a narrow tube with a camera) is inserted through the mouth (peroral) to cut the muscles near the pyloric sphincter (a myotomy). This helps permanently relax the sphincter, which allows food to empty freely into the small intestine [5].

The Gastroparesis Cardinal Symptom Index (GCSI) was developed to assess the hallmark symptoms of gastroparesis (see Table 4 in the Appendix) and represents a subset of the longer, 20-item questionnaire, which is the Patient Assessment of Upper Gastrointestinal Disorders Symptoms (PAGI-SYM) questionnaire, was developed to assess symptoms of gastroparesis, functional dyspepsia, and gastroesophageal reflux disease. It is a 6-point Likert scale from 0 (none) to 5 (very severe). Patients are instructed to answer the survey based on their symptoms over the prior two weeks. Symptoms are then averaged to calculate the GCSI score with a minimum score of 0 and a max of 5 [6].

Since the introduction of GPOEM as a treatment option for gastroparesis, multiple individual studies have been done to investigate its success. This systematic review aims to evaluate the cumulative data on the procedure's long-term clinical efficacy and safety.

## Review

Methods

Study Conduction

Prior to conducting the review, a protocol was developed. The study was conducted in line with the Preferred Reporting Items for Systematic Reviews and Meta-Analyses Checklist [7].

Search Strategy

The databases PubMed, Ovid, Embase, and Google Scholar were used to conduct a literature search.

Eligibility Criteria

Articles were eligible for inclusion in the review if they were prospective or retrospective studies with a GCSI score at baseline and another GCSI at least one-year follow-up. Studies were excluded if they were systematic reviews or meta-analyses that contained duplicate data on the same patient population, redo GPOEM, surgery for indications other than refractory gastroparesis, or were not in English. If information was missing, the authors were contacted by email for further clarification or additional information.

Data Screening

The title and abstract of all studies returned from the different databases were independently and blindly screened by the first two authors (TS and KS) using the Rayyan QCRI software package. The screening identified studies that met the inclusion criteria. The two authors resolved discrepancies during the screening process through discussion.

Data Extraction and Management

Two reviewers extracted all relevant data from the included studies using Microsoft Excel (Microsoft® Corp., Redmond, WA, USA). The data extracted had the first author's name, year of publication, the country the analysis was performed, the number of patients included in the study, the GCSI score at baseline, GCSI follow-up, the definition of clinical success, etiology of gastroparesis, percentage of patients who obtained clinical success, length of stay at the hospital, and post-operative adverse events.

Quality Appraisal

Two independent investigators (TS, KS) conducted a quality assessment by using the National Institutes of Health (NIH) quality assessment tool for before and after studies with no control group [8]. There were no discrepancies.

Results

**Figure 1:**
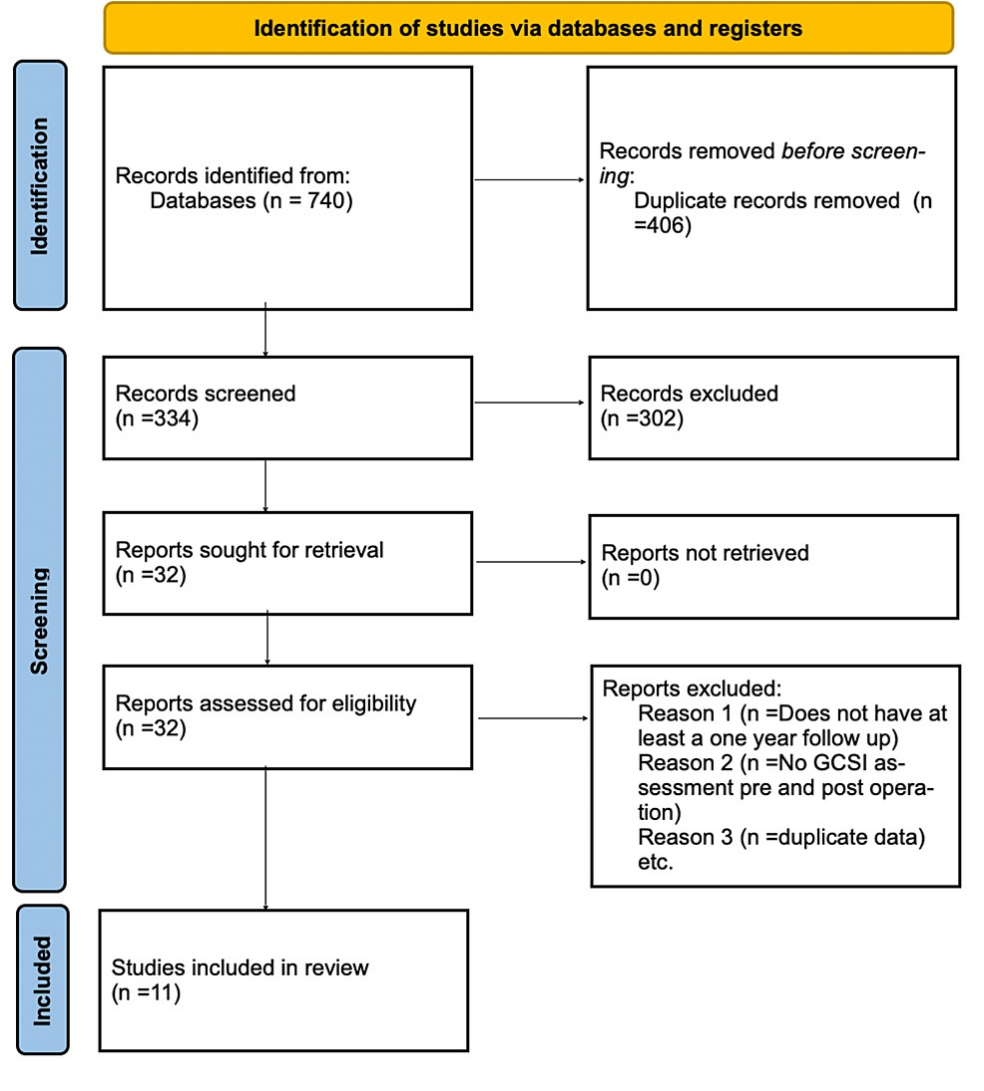
PRISMA Chart

**Figure 2:**
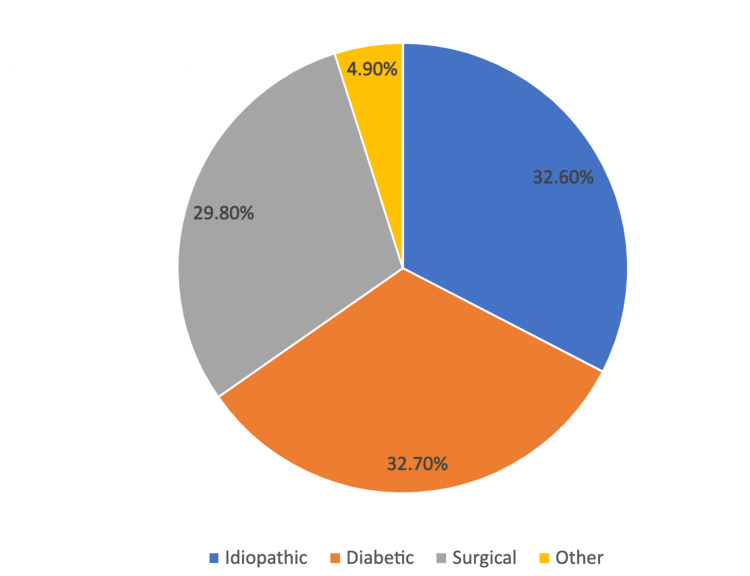
Etiologies of Gastroparesis

A total of 740 articles were found; of those, 32 were eligible for full-text review (Figure 1). After review, 11 articles were included (900 patients) in this paper. Studies were published from May 2017 to January 2022. Seven of the studies are retrospective, while four are prospective. Five studies are from the USA, two from France, one from the Czech Republic, one from the Netherlands, one from China, and one from Mexico. In 11 studies, 900 patients were treated for refractory gastroparesis, 65.8% were female (593 patients), and 34.2% were male (307 patients). Of the 900 patients, 294 had idiopathic, 295 had diabetic, 269 had post-surgical, and 44 had other for the etiology of gastroparesis (Figure 2). The mean age of patients was 42.4 to 55 years in nine studies. The median age of patients was 40 and 56 in two studies. All patients in the study had refractory gastroparesis.

Clinical Success

Eleven studies had a one-year follow-up, seven had a two-year follow-up, four had a three-year follow-up, and one had a four-year follow-up. An average decrease of GCSI by 1 point compared to baseline GCSI for all patients (described as clinical success) was found in 662 patients out of 713 (92.8%) at one-year follow-up, 421 out of 460 (91.5%) at two-year follow-up, 270 out of 270 (100%) at three-year follow-up, and 102 out of 102 (100%) at four-year follow-up (Table 1). Clinical success definitions differed among the studies. The range of clinical success according to each paper in year one was from 33-94.3%, year two from 50-89.9%, year three from 65.2-82.9%, and year four at 77.5% when evaluating each patient (Table 2).

**Table 1:** Changes in GCSI from baseline to follow-up time GCSI: Gastroparesis Cardinal Symptom Index

Study (Year)	Mean Pre- OP GCSI	Number of Patients at Baseline	Mean Post-OP GCSI at 1 Year	Number of Patients at 1 Year	Mean Post-OP GCSI at 2 Years	Number of Patients at 2 Years	Mean Post-OP GCSI at 3 Years	Number of Patients at 3 Years	Mean Post-OP GCSI at 4 Years	Number of Patients at 4 Years
Gregor et al. [9]	2.9 ± 0.9	76	2.0 ± 1.3	27	1.6 ± 1.4	14	1.8 ± 1.9	3		
Hernández Mondragón et al. [10]	3.84 ± 0.5	374	1.57 ± 0.4	331	1.56 ± 0.4	303	1.94 ± 0.7	214	2.21 ± 0.6	102
Labonde et al. [11]	3.33	46	1.85	46	1.80	46	1.85	46		
Conchillo et al. [12]	3.1 ± 0.1	24	2.4 ± 0.2	24						
Vosoughi et al. [13]	2.8 ± 1.1	80	1.5 ± 1.2	75						
Abdelfatah et al, [14]	3.8 ± 0.6	90	2.3 ± 1.3	48	1.6 ± 1.2	21	1.1 ± 1.2	7		
Tan et al. [15]	2.9 ± 0.6	79	1.04 ± 0.5	60	0.88 ± 0.4	33				
Hustak et al. [16]	3.16 ± 0.8	9	1.07 ± 0.6	9	1.31 ± 0.8	4				
Ragi et al. [17]	3.6	76	1.89	76	3.2	39				
Mekaroonkamol et al. [18]	3.49 ± 0.6	30	1.9 ± 1.2	11						
Dacha et al. [19]	3.41 ± 0.5	16	1.46 ± 1.4	6						

**Table 2:** Clinical success

Study (Year)	Clinical Success Definition	Percentage of Patients who Obtained Clinical Success
Gregor et al. [9]	A significant improvement in GCSI was defined as a decrease in at least 1 averaged point in the total GCSI score with more than a 25% decrease in at least 2 subscales	57.1% at 12 months, 50% at 24 months
Hernández Mondragón et al. [10]	A decrease of at least 1 point in the average total GCSI score with more than a 25% decrease in at least 2 subscales of cardinal symptoms	94.3% at 12 months, 89.9% at 24 months, 82.9% at 36 months, 77.5% at 48 months
Labonde et al. [11]	A decrease of at least 1 point in the GCSI compared with the preprocedural GCSI score	69.5% at 12 months, 69.5% at 24 months, 65.2% at 36 months
Conchillo et al. [12]	At least a 1-point decrease in the mean overall GCSI score	33% at 12 months
Vosoughi et al. [13]	At least 1 point decrease in the total GCSI scoring system with more than a 25% decrease in at least 2 of the subscales	56% at 12 months
Abdelfatah et al. [14]	Decrease of at least 1 point in the average total GCSI score with more than a 25% decrease in at least 2 subscales of cardinal symptom	69.1% at 12 months
Tan et al. [15]	Clinical response was defined as more than 25% decrease in at least 2 subscales of the GCSI scale	78.3% at 12 months, 81.8% at 24 months
Hustak et al. [16]	Treatment success was defined as a decrease of the total GSCI score of at least 40% from its baseline value	88.9% at 12 months, 75% at 24 months
Ragi et al. [17]	A decrease in the GCSI by at least 1 point compared to baseline	65.8% at 12 months, 74.4% at 24 months
Mekaroonkamol et al. [18]	A decrease in at least 1 averaged point in the total GCSI score with more than a 25% decrease in at least 2 subscales	57.1% at 12 months
Dacha et al. [19]	A decrease in mean GCSI, a significant decrease in at least 2 subsets of cardinal symptoms, and no hospitalization for gastroparesis	81% of patients at 12 months

Adverse Events

Adverse events occurred post-procedural in 62 out of 835 patients (in nine studies), with two of the most frequent being bleeding and mucosal tears - none of the adverse events led to mortality. The median length of stay post-operation ranged from one to two days, while the mean length ranged from 2.2 to 3.37 days (Table 3).

**Table 3:** Adverse events and hospital stay

Study (Year)	Length of Hospital Stay	Adverse Events and Number of Patients that Experienced Each One	Ratio of Adverse Event: No Adverse Event Post Operation
Gregor et al. [9]	NA	NA	NA
Hernández Mondragón et al. [10]	Median was 2 days	17 bleeding, 8 mucosal tear, 1 perforation, 2 clip dislodgement, 4 prepyloric ulcer	32:374
Labonde et al. [11]	Mean length 3.37 days	NA	NA
Conchillo et al. [12]	Mean 2.8 ± 0.3 days	2 pneumoperitoneum. 1 post-operative melena with no signs of active bleeding	3:24
Vosoughi et al, [13]	Median 1 day	3 capnoperitoneum, 1 mucosotomy, 1 thermal mucosal injury	5:80
Abdelfatah et al. [14]	Mean 2.2 ± 2 days	1 tension capnoperitoneum, 1 bleeding from the ulcer at the mucosotomy site, 1 abdominal pain, 1 exacerbation of pre-existing COPD	4:90
Tan et al. [15]	Mean 5.35 ± 0.8 days	7 suffered upper abdominal pain	7:79
Hustak et al. [16]	Mean is 2.9 days	1 insufficient mucosotomy closure, 1 gastrointestinal bleeding from a large ulceration at the site of the submucosal tunnel	2:9
Ragi et al. [17]	Median was 2 days	1 abdominal abscess, 2 delayed hemorrhage, 1 ulcer perforation, 2 functional occlusion, 1 persistent abdominal pain, 1 abdominal pain and fever	8:133
Mekaroonkamol et al. [18]	Mean 2.4 ± 1.0 days	1 tension capnoperitoneum without frank perforation or extraluminal leakage	1:30
Dacha et al. [19]	Mean 2.6 ± 0.7 days	No adverse events occurred	0:16

Discussion

Gastroparesis is a disease process that causes patients to have severe debilitating symptoms. Before GPOEM, the only other surgical option was a laparoscopic pyloroplasty. This systematic review evaluated the long-term efficiency of GPOEM. This systematic review evaluated eleven studies and provided evidence that GPOEM resulted in a modest decrease in the GCSI score. The reduction of GCSI score was sustained for one, two, three, and even four years post-operation. On average, after two years post-operation, the GCSI score decreased by at least one point. This is the first review that looks at the long-term benefit of GPOEM. Another systematic review looked at the early outcomes (mean follow-up of 7.8 months) in 292 patients, which also yielded the same improvement in GCSI [20]. In this study, a mean GCSI score of 3.3 ± 0.6 dropped to 1.61 ± 0.61 after one year. This one-point drop in GCSI meets our definition of clinical success in this systematic review.

Clinical success varied among the studies and can be attributed to multiple factors. The definition of clinical success was not standardized among the studies. With such variability in definitions and reporting of study data, the only uniform clinical definition that could be applied was the one-point drop in GCSI, which was used in the studies by Conchillo, Lablonde, and Ragi. The improvement of a decrease of more than one point has been shown to be clinically relevant. Individual symptoms could not be evaluated as only some papers had included this in their study. Overall, most patients found relief of symptoms having the GPOEM after one year. However, an outlier was observed in the study done by Conchillo, in which a 33% success rate was reported. The study explains this as unclear, but possibly due to differences in patient characteristics. After two years, in six of the seven studies, the patients met our clinical success definition. However, in one study by Ragi, the average baseline GCSI score was 3.6. After one year, this decreased to 1.89; after two years, it had increased to 3.2. This study explains this increase as no previous study has looked at such an extended follow-up and preoperative factors that predict the procedure's success should be evaluated to find what patient demographic would most benefit from the GPOEM for long-term symptom relief. The studies that had three years (all four studies) and four years (one study) met our definition of clinical success.

Adverse events that occurred postoperatively were 7% of the time. The most common were bleeding and mucosal tears. Long-term events were not listed in the papers. In the study done by Ragi, the total number of patients who had the GPOEM procedure performed was 133, but only 76 patients were evaluated for GCSI symptoms [17].

In this systematic review, other variables such as length of hospital stay and adverse events show that GPOEM is a relatively safe option compared to laparoscopic pyloroplasty. One study found that for GPOEM, the average hospital stay was 1.4 days, and adverse events occurred 3.3% of the time. In contrast, to laparoscopic pyloroplasty, the average hospital stay was 4.44 days, and adverse events occurred in 16.7% of patients. This systematic review of the GPOEM has similar results regarding the length of hospital stay for the GPOEM (median, 1 to 2 and mean, 2.2 to 3.37 days) [21].

The present study has several limitations. First, not all patients who were part of the baseline cohort continued with the follow-up throughout the study. This may lead to bias, as unhappy patients may withdraw from the study, while patients with satisfactory results are willing to participate. Secondly, this review was done by two authors, which could have led to some bias when deciding whether to include or exclude an article.

## Conclusions

In conclusion, patients with refractory gastroparesis who undergo the GPOEM, have a long-term decrease in the symptoms associated with gastroparesis. In conclusion, patients with refractory gastroparesis who undergo the GPOEM have a long-term reduction in the symptoms associated with gastroparesis. In addition, the procedure has a small percentage of complications and short-term hospital stay. Future studies could further evaluate if the etiology of gastroparesis affects success.

## Appendices

**Table 4:** Gastroparesis Cardinal Symptom Index (GCSI) Adapted from [22].

	None	Very Mild	Mild	Moderate	Severe	Very Severe
1. Nausea (feeling sick to your stomach as if you were going to vomit or throw up)	0	1	2	3	4	5
2. Retching (heaving as if to vomit, but nothing comes up)	0	1	2	3	4	5
3. Vomiting	0	1	2	3	4	5
4. Stomach fullness	0	1	2	3	4	5
5. Not able to finish a normal-sized meal	0	1	2	3	4	5
6. Feeling excessively full after a meal	0	1	2	3	4	5
7. Loss of appetite	0	1	2	3	4	5
8. Bloating (feeling like you need to loosen your clothes)	0	1	2	3	4	5
9. Stomach or belly visibly large	0	1	2	3	4	5
